# Amplitude Modeling of Specular Multipath Components for Robust Indoor Localization

**DOI:** 10.3390/s22020462

**Published:** 2022-01-08

**Authors:** Hong Anh Nguyen, Van Khang Nguyen, Klaus Witrisal

**Affiliations:** 1 School of Electronics and Telecommunications, Hanoi University of Science and Technology, Hanoi 11615, Vietnam; khang.nguyenvan1@hust.edu.vn; 2 Signal Processing and Speech Communication Lab, Graz University of Technology, 8010 Graz, Austria; witrisal@tugraz.at

**Keywords:** situational awareness, environmental awareness, location awareness, geometry-based channel modeling, gaussian process regression, single-anchor positioning, multipath-assisted positioning, UWB radios, mm-wave radios

## Abstract

Ultra-Wide Bandwidth (UWB) and mm-wave radio systems can resolve specular multipath components (SMCs) from estimated channel impulse response measurements. A geometric model can describe the delays, angles-of-arrival, and angles-of-departure of these SMCs, allowing for a prediction of these channel features. For the modeling of the amplitudes of the SMCs, a data-driven approach has been proposed recently, using Gaussian Process Regression (GPR) to map and predict the SMC amplitudes. In this paper, the applicability of the proposed multipath-resolved, GPR-based channel model is analyzed by studying features of the propagation channel from a set of channel measurements. The features analyzed include the energy capture of the modeled SMCs, the number of resolvable SMCs, and the ranging information that could be extracted from the SMCs. The second contribution of the paper concerns the potential applicability of the channel model for a multipath-resolved, single-anchor positioning system. The predicted channel knowledge is used to evaluate the measurement likelihood function at candidate positions throughout the environment. It is shown that the environmental awareness created by the multipath-resolved, GPR-based channel model yields higher robustness against position estimation outliers.

## 1. Introduction

The vision of an Internet-of-Things (IoT) has been discussed extensively, in recent years, covering end-user applications in a wide range of application domains, including industrial [1], health-care and assisted living [2], smart cities, and smart mobility [3]. Similarly, 5G and beyond-5G wireless networks are currently under development, aiming for instance at an end-to-end latency in the range of 1 ms, high user densities, and unprecedented data rates per volume for a heterogeneous set of end-user scenarios [4,5,6]. Location information is a critical component of many of these applications [7], creating context-awareness with different service quality requirements [8]. The relevant performance criteria extend far beyond the required level of accuracy. For assisted and autonomous driving, for example, location information is of mission-critical importance, making it a prime example of a high-end location-based application with a need for 100% availability [9,10,11,12]. Robustness, security, and privacy issues in IoT applications have recently been discussed in [13]. High-accuracy location information is seen as a key enabler for location-based services in the IoT and in 5G networks [12,14].

Much work has been done to address the various issues involved in accurate and robust position estimation, demonstrating the heterogeneity of scenarios and applications as discussed before. RSS-based techniques are very suitable for positioning based on signals-of-opportunity, because it does not require dedicated time stamping and synchronization. RSS measurements can be used to compute the position from path-loss-based range estimates [15,16], from ray-tracing based propagation predictions [17,18], from fingerprinting/machine-learning-based methods [18,19,20], and from tomography-inspired, device-free measurements [21]. The main disadvantage of this approach is the low accuracy and low robustness compared with other measurement principles [22], in particular, when multipath fading and shadowing are taken into account.

Time-of-flight (ToF) measurements and multilateration are usually the basis for robust, high-accuracy positioning systems, including global navigation satellite systems (GNSS) [23]. It is well known that the ToF *ranging* error variance scales reciprocally with the squared bandwidth and the signal-to-noise ratio (SNR) [24], which motivates the advantage of having a high signal bandwidth available. The SNR depends only weakly on the range (ensured e.g., due to power control mechanisms in the network), however, multipath will have a severe influence [25,26,27,28]. These basic insights have been used to study the *positioning* performance of wireless networks [26,29], showing the influence of the geometry (and number) of the nodes that are being involved in the position computation.

The availability of sufficiently accurate location information is a facilitator for exploiting “location awareness” to enhance the performance of the radio access network [30]. Location-awareness leverages geometric information to optimize algorithms and protocols of a wireless communication system at different layers of the protocol stack, to satisfy the needs for higher throughput, lower latency, higher robustness, and other performance indicators [30]. At higher layers of the stack, location information has been employed directly for routing algorithms [31,32]. At lower layers, a channel quality metric (CQM) is often introduced, which maps between a network performance indicator, e.g., the channel capacity or the signal-to-interference-plus-noise ratio (SINR) and the position, yielding a so-called “radio environment map” (REM) [33]. The REM is obtained from a database, which is acquired from measurements conducted beforehand. Crowd-sensing has been proposed for the data collection, where data covering a wide spatial area is being collected by a large number of agents navigating through an environment [34]. A regression tool is needed to handle the large database and perform predictions. Gaussian process regression (GPR) is of particular interest because it enables the development of inference algorithms whose complexity does not grow with the number of observations [34,35].

In [36,37], situational awareness has been defined, a concept where the location information of radio nodes is collected together with information about the propagation environment. The environment model is comprised of specular multipath components (SMCs), their angle-of-arrivals (AOAs), angle-of-departures (AODs), the corresponding delays as well as points of reflections. This information can be deduced from channel measurements by means of a Simultaneous Localization And Mapping (SLAM) algorithm [38,39], where SMCs are modeled by virtual anchors (VAs), i.e., mirror images of the anchor w.r.t. different reflecting surfaces. Using these VA positions together with a data association algorithm, multipath-assisted, single-anchor localization and tracking algorithms can be formulated. Such algorithms exploit the VA-based environment model, achieving centimeter accuracy when UWB signals are used [14,38,40,41]. Crowd-sourcing of such map information has been studied in [42]. Situational awareness is of specific interest for the beam-prediction in mmWave radio systems [37].

In this paper, we combine the VA-based geometric environment model and a GPR model. The former is used to predict delay, AOA, and AOD of SMCs, while the latter is used to predict the amplitudes of SMCs, as described in [43]. Extending over our previous paper [43], the following main contributions are made:

- We formulate a REM that predicts the SMC amplitudes, the SINR of SMCs, and the position error bound (PEB) at any position throughout the whole floor plan.
- We analyze a database of UWB radio channel measurements to show to what extent the REM represents the multipath components in this set of measured channel impulse response (CIR) data. To this end, we study the number of resolvable SMCs and the energy capture (EC) of these SMCs, which is the fraction of energy carried by the SMCs in relation to the total energy of the CIR.
- We show the usefulness of the REM in improving the robustness of a multipath-based single-anchor localization algorithm.

The structure of the paper is as follows. The UWB indoor signal model and geometry-based channel model are described in Section 2. The parameters represented by the REM, the SMC amplitudes, the SINRs, and the PEB, are also formulated. The experimental data, as well as data pre-processing steps, are described in Section 3. Section 4 discusses the Gaussian process model (GPM) based channel predictions for the above REM. Section 5 shows how the REM is exploited for a multipath-based single-anchor localization algorithm. Section 6 shows the localization results. Section 7 concludes the paper.

## 2. System and Environment Model

### 2.1. System Model

**Figure 1:**
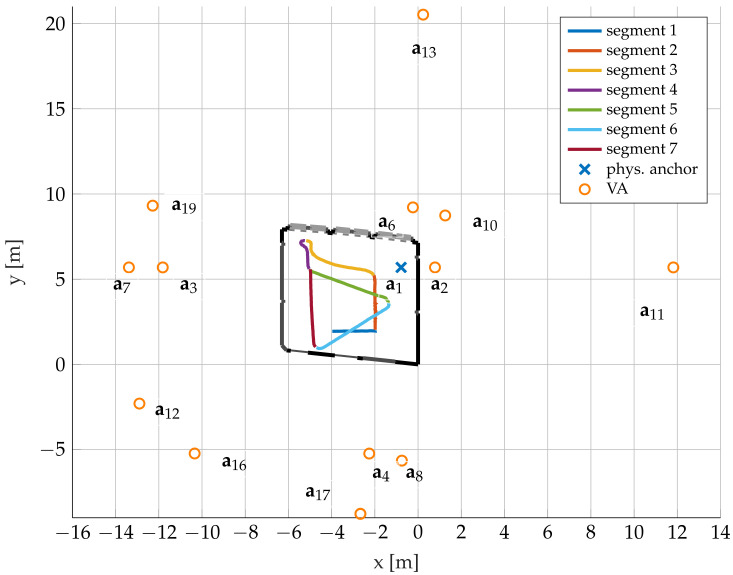
Floor plan of the evaluation scenario. Bold black lines denote walls, thick gray lines represent glass windows, other lines illustrate other materials. One blue cross represents the PA; orange circles denote VAs which were considered in the experimental evaluation. An agent moves along a trajectory segmented into seven parts indicated with distinct colors.

We consider an indoor wireless communication environment with a fixed physical anchor (PA) at position $a_{1}$, as illustrated in Figure 1, and a mobile agent at position p. The agent is moved along a segmented trajectory. Some VAs $a_{k}$ up to order two are also shown, i.e., mirror images of the PA at the corresponding flat surfaces. The VAs establish a geometric model of SMCs originating from these surfaces. The VA-model can be used as a deterministic description of the delay, AOA, and AOD of these SMCs, given the position of the agent.

We describe the radio channel from the PA located at position $a_{1}$ and the agent node located at position p via a geometry-based stochastic channel model by:

(1) $$h\left(\tau ;p\right)=\sum_{k=1}^{K}\alpha_{k}\left(p\right)\delta \left(\tau -\tau_{k}\left(p\right)\right)+\nu \left(\tau ;p\right)$$

**Figure 2:**
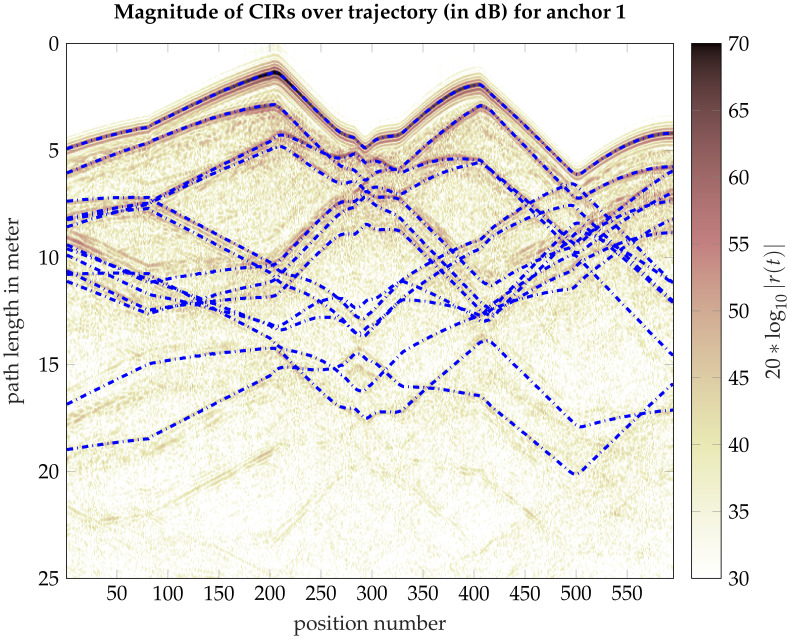
Measured CIRs over the trajectory at a bandwidth of 2 GHz. The blue dash lines indicate expected paths of the SMCs corresponding to VAs up to order two, estimated using the SLAM algorithm from [38,45] and refined as described in [46]. The locations of VAs are shown in Figure 1.

The first term on the RHS of (1) is the sum of *K* SMCs with amplitude $\alpha_{k}$ and delay $\tau_{k}$ described as functions of the mobile agent position p. The delay $\tau_{k}\left(p\right)$ corresponds to the distance between the agent and the PA (for k=1) or VA (for k=2,…,K). That is $\tau_{k}\left(p\right)=∥p-a_{k}∥/c$, where $a_{k}$ is the position of the respective PA or VA, and *c* is the speed of light. In Figure 2, the path lengths $d_{k}\left(p\right)=∥p-a_{k}∥$ w.r.t various VAs at positions $a_{k}$ are plotted on top of the measured CIRs. The second term denotes the diffuse multipath (DM) ν(τ;p), modelled as a zero-mean Gaussian random process. We assume uncorrelated scattering along the delay axis, hence the autocorrelation function of ν(τ;p) is given by:

(2) $$K_{\nu }\left(\tau ,u;p\right)=E\left\{\nu \left(\tau ;p\right)\nu^{*}\left(u;p\right)\right\}=S_{\nu }\left(\tau ;p\right)\delta \left(\tau -u\right),$$

where $S_{\nu }\left(\tau ;p\right)$ is the power delay profile (PDP) of the DM at agent position p. The DM process is assumed to be quasi-stationary in the spatial domain, i.e., $S_{\nu }\left(\tau ;p\right)$ does not change in the vicinity of p [44].

A base band signal s(t) is transmitted at carrier frequency $f_{c}$. The complex envelope of the received signal then read:

(3) $$r\left(t;p\right)=\sum_{k=1}^{K}\alpha_{k}\left(p\right)s\left(t-\tau_{k}\left(p\right)\right)+\left(s*\nu \right)\left(t;p\right)+w\left(t\right)$$

The third term of (3) is the measurement noise w(t) modelled as additive white Gaussian noise (AWGN) with double-sided power spectral density $N_{0}/2$. To use (3) for estimating parameters of SMCs, it is useful to minimize autocorrelation side lobes of s(t). A root-raised-cosine (RRC) pulse is thus considered for the transmitted signal with pulse duration $T_{s}$. The energy of s(t) is normalized to one, i.e., $\int {\left|s(t)\right|}^{2}dt=1$.

### 2.2. Position Error Bound

The channel model in (1) has been used in [27,38,47,48] to analyze multipath-assisted indoor positioning systems and to develop algorithms for it. In [27,48], the PEB has been derived for the signal model. It quantifies how the delay information of each SMC contributes useful information that can be exploited by a positioning algorithm. In this paper, the PEB is modeled as a spatial field for the current application environment, providing a measure for the achievable positioning accuracy in the given scenario. To develop this “environment model”, first, the PEB is explained in detail.

The PEB for the multipath-assisted positioning problem is the Cramer-Rao lower bound on the position error, the square-root of the trace of the upper left 2-by-2 submatrix of the inverse Fisher information matrix (FIM) $J_{\theta }$ [48]

(4) $$P\left\{p\right\}=\sqrt{\mathrm{tr}\left\{{\left[J_{\theta }^{-1}\right]}_{2\times 2}\right\}}=\sqrt{\mathrm{tr}\left\{J_{p}^{-1}\right\}}$$

where $\theta ={\left[p^{T}{R\alpha }^{T}{I\alpha }^{T}\right]}^{T}$ is a parameter vector containing the unknown location of the mobile agent, and the real and imaginary parts of the SMC complex amplitudes. $J_{p}$ is the equivalent FIM (EFIM) [26]. According to [48], we have

(5) $$J_{p}=\frac{8\pi^{2}\beta^{2}}{c^{2}}\sum_{k=1}^{K}{\mathrm{SINR}}_{k}\left(p\right)J_{r}\left(\phi_{k}\right).$$

in which $\beta^{2}=\int f^{2}{\left|S(f)\right|}^{2}df$ is the effective bandwidth (S(f) is the Fourier transform of s(t)) and *c* is the speed of light. The summation shows that the information from individual SMCs is added up. Each SMC contributes information proportional to the ${\mathrm{SINR}}_{k}$ and the ranging direction matrix $J_{r}\left(\phi_{k}\right)$ [26]. It is obvious that the more SMC information is available, the lower the PEB. The ${\mathrm{SINR}}_{k}\left(p\right)$ is defined as

(6) $${\mathrm{SINR}}_{k}\left(p\right)=\frac{{|\alpha_{k}\left(p\right)|}^{2}}{N_{0}+T_{p}S_{\nu }\left(\tau_{k};p\right)}$$

with $T_{p}$ being the effective pulse duration [43]. It quantifies the interference of the DM, expressed by the PDP $S_{\nu }\left(\tau_{k};p\right)$, onto the *k*-the SMC with energy $|\alpha_{k}{\left(p\right)|}^{2}$. The ranging direction matrix describes the geometry of the setup. The delay information of the *k*-th SMC is directed towards the *k*-th VA, expressed by

(7) $$J_{r}\left(\phi_{k}\right)=\left[\begin{array}{cc} \cos^{2}\left(\phi_{k}\right) & \cos\phi_{k}\sin\phi_{k}\\ \cos\phi_{k}\sin\phi_{k} & \sin^{2}\left(\phi_{k}\right) \end{array}\right]$$

where $\phi_{k}$ is the angle from the corresponding VA to the mobile agent at position p.

From (6) it can be seen that the range information contributed by the *k*-th SMC is quantified by ${\mathrm{SINR}}_{k}\left(p\right)$. Knowledge of ${\mathrm{SINR}}_{k}\left(p\right)$, therefore, allows to properly select and weigh the SMCs to be used by positioning algorithms. The idea of this paper is to model the ${\mathrm{SINR}}_{k}\left(p\right)$ of the SMCs as a spatial field in order to obtain a map of the expected PEB for the current application environment. Such an “environment map” will provide the side information needed to make a positioning algorithm more robust.

### 2.3. Radio Environment Map Using Gaussian Process Regression

According to [34], a spatial field is the distribution of a physical quantity such as RF interference, pollution, temperature, humidity, light intensity, etc., which, if known, will foster the development of a new context-aware application and algorithms. In the context of radio systems, these spatial distributions are referred to as *radio environment maps* (REMs).

In this paper, we are interested in investigating the characteristics of individual SMCs, i.e., the direct link and the ones that resulted from reflections on flat surfaces. Spatial fields are obtained from mapping the agent positions with these SMC characteristics. In the UWB communication system, thanks to its very high time resolution, these SMCs can be resolved and predicted as studied in [43].

The SMC characteristics that are modeled in this paper are the SMC amplitudes and variances, thus the SINRs and the PEB can be predicted using (5) and (6). In Section 5, this information will be exploited to enhance the robustness of a multipath-assisted positioning algorithm. The SMC amplitudes and variances can also be used to predict the channel capacity and bit-error rate. The individual SMC metrics can then be useful for efficient beam selection and beam finding in mmWave wireless systems, exploiting prior information on potentially useful SMCs.

To construct the REM for the PEB, we need a spatial field model for the SMC amplitudes and the interference power of DM, c.f. (6). A Gaussian Process model (GPM) is used to learn such an amplitude model from observed data, as described in [43]. In a training phase, the SMC amplitudes ${\hat{\alpha }}_{k}\left(p\right)$ are estimated from the CIR. Given the known positions of the VAs and the agent, the expected delays $\tau_{k}\left(p\right)=\frac{1}{c}∥a_{k}-p∥$ are deduced, yielding

(8) $${\hat{\alpha }}_{k}\left(p\right)=\langle s\left(t-\tau_{k}\left(p\right)\right),r\left(t;p\right)\rangle .$$

Next, the distance dependence is removed from the SMC amplitudes, yielding the normalized amplitude data $\gamma_{k}\left(p\right)={\hat{\alpha }}_{k}\left(p\right)∥a_{k}-p∥\exp\left(j2\pi \frac{f_{c}}{c}∥a_{k}-p∥\right)$, which is assumed to depend on the direction angle $\phi_{k}\left(p\right)=∠\left(a_{k}-p\right)$, only, describing the antenna pattern and the reflectivity of the reflecting surface [43]. This direction dependence is modeled by means of GPR. Several dataset are obtained, i.e., $D_{k}={\left\{\phi_{k}\left(p\right),\gamma_{k}\left(p\right)\right\}}_{p\in P}$, where P is the set of measured positions. Please note that whenever there are overlapping SMCs detected within a CIR, the corresponding amplitudes are neglected/discarded.

In this paper, the dataset $D_{k}^{\mathrm{abs}}={\left\{\phi_{k}\left(p\right),\left|\gamma_{k}\left(p\right)\right|\right\}}_{p\in P}$ is used, which deals with only the SMC amplitude’s abscissa. According to [43], the hyper-parameters of the GPM are $\theta_{k}^{\mathrm{abs}}={\left[a_{k}^{\mathrm{abs}},\sigma_{k}^{\mathrm{abs}},\sigma_{\nu ,k}^{\mathrm{abs}},\beta_{k}^{\mathrm{abs}}\right]}^{T}$, in which $a_{k}^{\mathrm{abs}}$ is the coherence angle, $\sigma_{k}^{\mathrm{abs}}$ is the standard deviation of the correlation kernel, $\sigma_{\nu ,k}^{\mathrm{abs}}$ is the standard deviation accounting for the DM, and $\beta_{k}^{\mathrm{abs}}$ is the mean of the abscissa of the k-th SMC amplitude.

From these data sets and hyper-parameters $\theta_{k}^{\mathrm{abs}}$ of the GPM, one can estimate the mean $E[\psi \left(\phi_{k}\left(p^{*}\right)\right)|D_{k},\theta_{k}^{\mathrm{abs}}]$ and variance $V[\psi \left(\phi_{k}\left(p^{*}\right)\right)|D_{k},\theta_{k}^{\mathrm{abs}}]$ of the normalized SMC amplitudes at any agent position of interest $p^{*}$ [43], and hence the ${\mathrm{SIN}R}_{k}\left(p^{*}\right)$ and the PEB $P(p^{*})$, which are the REMs investigated in this paper. Specifically, we get

(9) $${\mathrm{SIN}R}_{k}\left(p^{*}\right)\approx \frac{E^{2}\left[\psi \left(\phi_{k}\left(p^{*}\right)\right)|D_{k},\theta_{k}^{\mathrm{abs}}\right]}{2V[\psi \left(\phi_{k}\left(p^{*}\right)\right)|D_{k},\theta_{k}^{\mathrm{abs}}]},$$

c.f. Appendix B in [43]. Note that the predicted ${\mathrm{SIN}R}_{k}\left(p^{*}\right)$ is independent of the anchor-agent distance $∥a_{k}-p^{*}∥$.

Section 4 shows how the spatial fields are learnt. Section 5 and Section 6 show how this information is utilized for the single-anchor multipath-based localization algorithm.

## 3. Experiment

### 3.1. Experiment Setup

An experiment was conducted in a laboratory room at the Graz University of Technology, as illustrated in Figure 1. The room consists of two plasterboard walls and two reinforced concrete walls (shown as black outer lines), three glass windows at the north wall (shown as thick gray lines), one whiteboard, and one wooden door at the south wall. In the following sections, we label these walls by east plasterboard (EPB), south wall (SW), west wall (WW), and north glass window (NGW), respectively.

**Figure 3:**
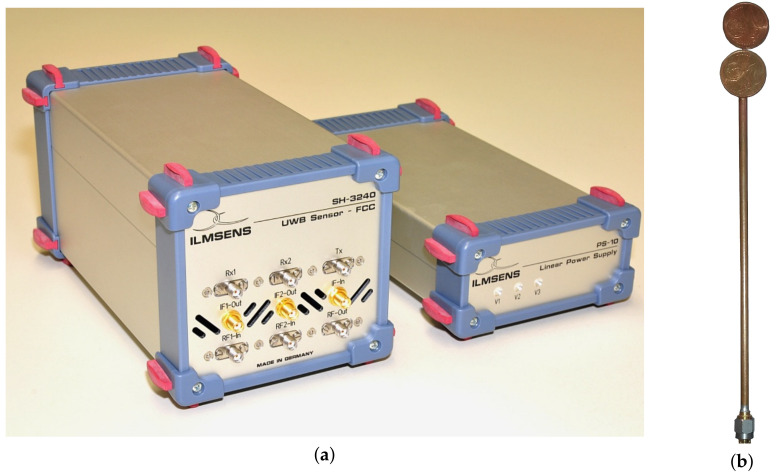
Equipments used for the experiment: (**a**) Ilmsens UWB M-sequence sounder and Ilmsens power supply, (**b**) coin antenna.

A measurement data set was acquired with an Ilmsens Ultra-Wideband M-sequence channel sounder [49], c.f. Figure 3a, which provides a transmitted signal with 6.95 GHz carrier frequency to acquire a frequency band between approximately 3.5 and 10.5 GHz. The measurement principle is correlative channel sounding [50], in which a binary-code sequence with suitable correlation properties are transmitted and correlation with the known-code sequence is performed at the receiver to obtain the CIR.

Dipole coin antennas, shown in Figure 3b, have been used for the measurements. According to [51], the coin antenna has a very wide bandwidth ranging from 3 to 9 GHz and a nearly isotropic radiation pattern in the horizontal plane. As shown in Figure 1, a receive antenna was placed at a fixed position $a_{1}$, whereas a transmit antenna (at position p) was moved along a trajectory segmented into 7 parts for easier interpretation. Both antennas were mounted on tripods at the same height, therefore only the co-polarized, azimuth radiation pattern of the antenna has an impact on the data. The raw measurements at the receiver port were filtered with an RRC pulse with center frequency 6.95 GHz, roll-off factor 0.5, and bandwidth $1/T_{p}=2$ GHz to obtain the received signals corresponding to the model in (3).

### 3.2. Experiment Pre-Processing

For the experiment, the exact positions of the mobile agent and the VAs were unknown. Thus, in a pre-processing step, we used the SLAM algorithm [38,45] and CIRs from the measurement to estimate the trajectory positions {p} and the locations of the VAs $\left\{a_{k}\right\}$ and PAs [43].

Different VAs can be distinguished from different traces of the path lengths, as illustrated in Figure 2.

Compared with [43], more VA positions are explored in this paper. The estimated trajectory and VAs are plotted in Figure 1. VAs 2, 3, 4, and 6 represent single reflections on the EPB, WW, SW, and NGW respectively. VAs 7, 8, 10, 11, 12, 13, 16, 17, and 19 represent double reflections on EPB then WW, EPB then SW, EPB then NGW, WW then EPB, WW then SW, SW then NGW, SW then WW, NGW then SW, and NGW then WW respectively.

Given the estimated positions of VAs $\left\{a_{k}\right\}$, and the receiver positions {p}, the expected delays $\tau_{k}\left(p\right)$ are computed. Next, the SMC amplitudes ${\hat{\alpha }}_{k}\left(p\right)$ are estimated from the CIR using (8). Finally, the distance dependence will be removed from the SMC amplitudes, yielding the normalized amplitude data $\psi (\phi_{k}\left(p\right))$, where $\phi_{k}\left(p\right)=∠\left(a_{k}-p\right)$ is the direction angle. Several dataset $D_{k}=\left\{\phi_{k}\left(p\right),\psi \left(\phi_{k}\left(p\right)\right)\right\}$ are then obtained.

On the basis of these data sets, the GPM is learned, using the built-in Matlab function *fitrgp* (Matlab version R2018a), yielding the hyper-parameters $\theta_{k}^{\mathrm{abs}}={\left[a_{k}^{\mathrm{abs}},\sigma_{k}^{\mathrm{abs}},\sigma_{\nu ,k}^{\mathrm{abs}},\beta_{k}^{\mathrm{abs}}\right]}^{T}$. From these and the data, prediction of the SMC amplitudes is performed using the function *predict*. Other spatial field indicators, such as SINR, PEB are then deduced, c.f. (4) and (6). The resulting REM will be discussed in Section 4, and exploited in Section 5 and Section 6.

## 4. Analysis of the GPR-Based Channel Model

In this section, various REM parameters, i.e., SINR, PEB, etc will be analyzed, using the measurement data. We first analyze the energy “captured” by the SMCs in relation to the total energy of the observed CIRs, denoted as energy capture (EC) [52] and the number of visible SMCs along the trajectory. Secondly, the GPM of the SMC amplitudes and the resulting REMs for SMC amplitudes, SINR-values, and the PEB are illustrated.

### 4.1. Energy Capture

**Figure 4:**
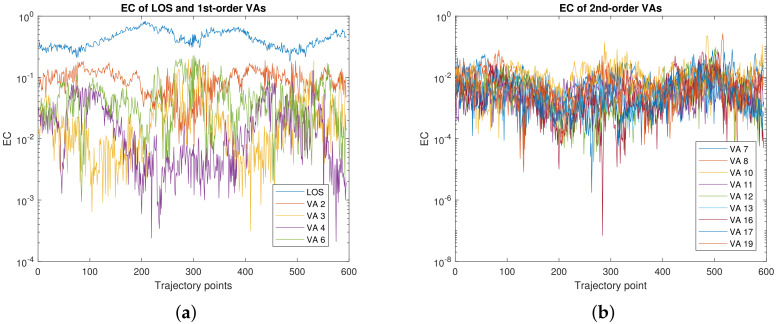
EC of individual SMC: (**a**) VA 1 to VA 6, (**b**) VA 7 to VA 19.

**Figure 5:**
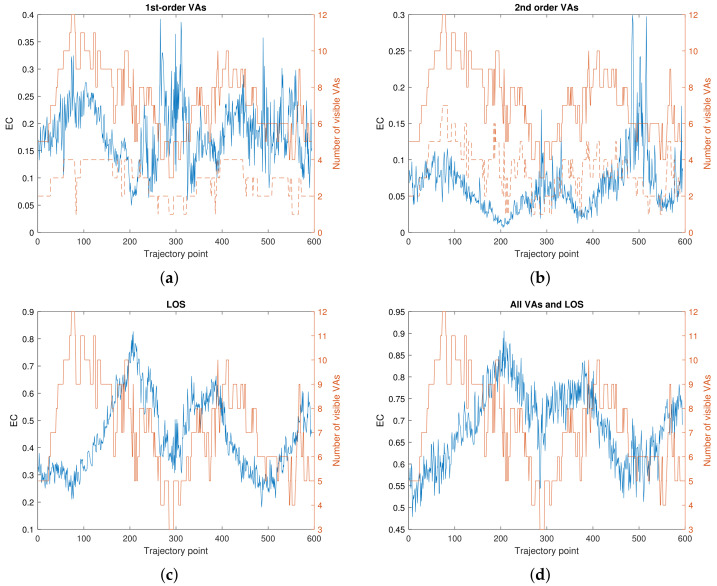
EC of certain SMCs vs. number of VAs visible: (**a**) first-order VAs only, (**b**) second-order VAs only, (**c**) LOS (PA) only, (**d**) all VAs and LOS (PA). The brown solid line shows the number of VAs that are visible from the corresponding trajectory point. The dashed brown line shows the number of visible VAs that are within the set of (**a**) first-order VAs or (**b**) second-order VAs.

We analyze the EC of the SMCs, which is defined as the energy ratio between one individual or a few SMCs over the total energy of the CIR [52]. Figure 4 plots the EC of individual SMCs, while Figure 5 shows the combined ECs of different sets of SMCs. The LOS component captures between 20% to 80% of the total received signal power; the closer the agent to the anchor, the higher the EC. Individual first-order SMCs capture up to 20% of the total received power, while the accumulated EC of all first-order SMCs is between 5% to 35%. For second-order SMCs, the ECs are between 0.1% to 5%, except for VA 8 and 10 where the maximum EC is 25% and 20% respectively. Their accumulated EC is mostly between 2% to 20%.

Figure 5 also plots the number of visible VAs at each trajectory point. For first-order and especially second-order SMCs, the accumulated EC correlates with the number of visible VAs, as shown in Figure 5a,b. It could be because for second-order VAs, the energy are relatively the same, as shown in Figure 4b. For first-order VAs, the energies for different VAs are quite different.

Figure 5c plots the EC of the LOS signal versus the total number of visible VAs. It is shown that the closer the PA to the anchor, the higher the EC and the fewer the number of visible VAs, which are both understandable.

Figure 5d illustrates the EC of all SMCs versus the number of visible VAs. It is seen that, for our data set, between 50% and 90% of the received power can be modeled by means of SMCs. Correspondingly, between 50% and 90% of the received power can be predicted accurately by means of a GPR amplitude model. A beam selection and beam searching algorithm can select strong first and second-order SMCs as candidate beams, in case the LOS gets blocked (e.g., by the user itself).

### 4.2. SMC Amplitudes and SINR

**Figure 6:**
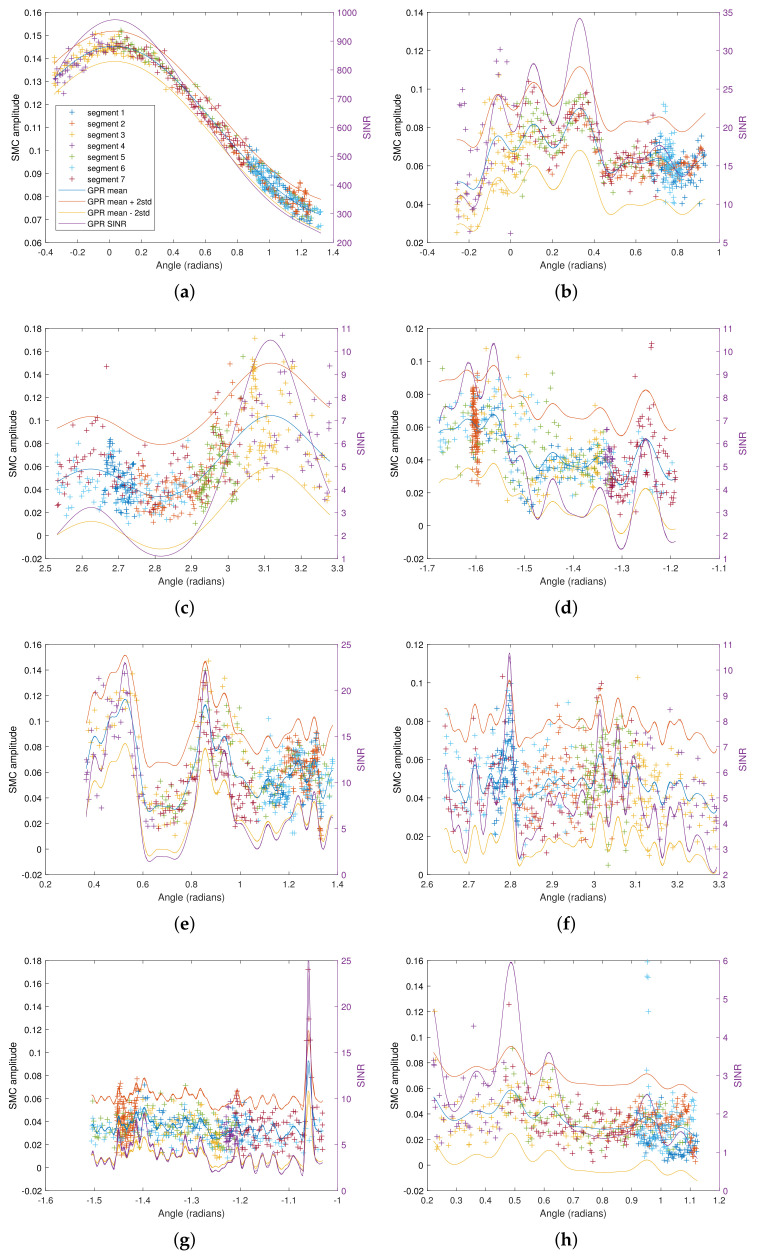

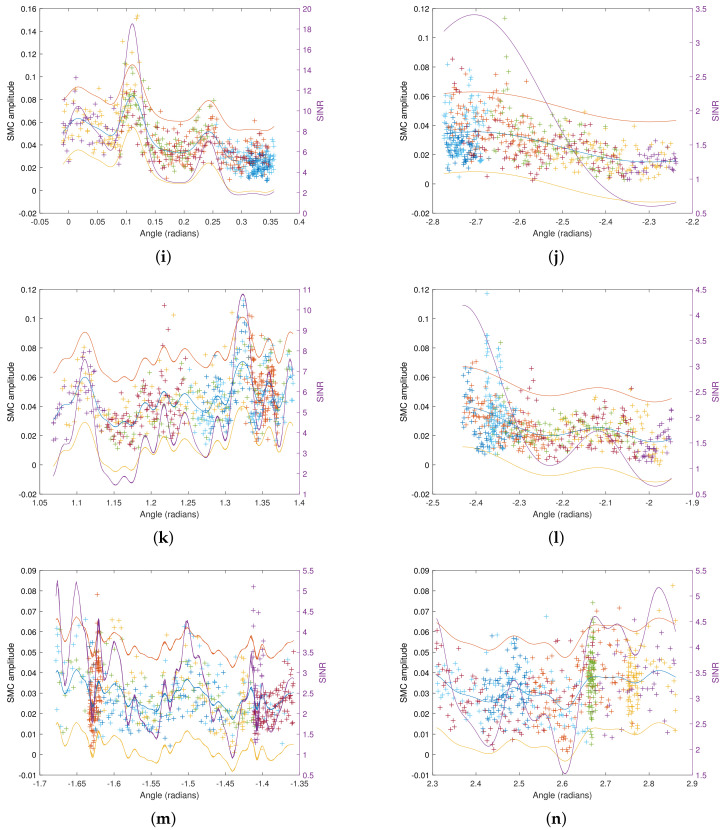
GPR on SMC amplitudes corresponding to: (**a**) LOS link, (**b**) reflection on EPB, (**c**) reflection on WW, (**d**) reflection on the south part of the room containing various materials, e.g., white board, wall, metal door, (**e**) reflection on the north part of the room, containing glass windows and walls, (**f**) double reflection on EPB then WW, (**g**) double reflection on EPB then SW, (**h**) double reflection on EPB then NGW, (**i**) double reflection on WW then EPB, (**j**) double reflection on WW then SW, (**k**) double reflection on SW then NGW, (**k**) double reflection on SW then WW, (**l**) double reflection on SW then WW, (**m**) double reflection on NGW then SW, and (**n**) double reflection on NGW then WW. The regressed amplitude is $|\alpha_{k}\left(p\right)d_{k}\left(p\right)|$. SINR computed from GPR is plotted in purple with axis on the right-hand side.

Figure 6 shows the GPR for various normalized SMC amplitudes $|{\hat{\alpha }}_{k}\left(p\right)d_{k}\left(p\right)|$ as a function of the direction angle $\phi_{k}\left(p\right)$ of the propagation path. The estimated amplitudes from the data set are shown along the mean and the ±2σ interval, as well as the predicted ${\mathrm{SINR}}_{k}\left(\phi_{k}\left(p^{*}\right)\right)$ given by (9). It is evident that there is a correlation between the SMC amplitudes and the direction angle, as described in Section 2.3, which is exploited by the GPR. The normalization does not allow for a direct comparison of the SMC amplitudes. But the comparison of the SINR-values indicates how much ranging information each of the SMCs can yield, c.f. (5).

It is shown that the SINR for reflections on the EPB is high, around 40, see Figure 6b which is much higher than the SINR for other SMCs, see Figure 6c–n, except the LOS link. Please note that on the east side of the room, there are fewer obstacles which makes the SMCs more reliable than others.

For the reflections on the WW, c.f. Figure 6c, the SINR is very low, indicating low ranging information, except at a direction approximately perpendicular to the wall, where the SINR increases steadily. The result is reasonable since, near the west wall, there are quite a few obstacles, e.g., computers, measurement equipment, etc., that could produce scattering effects.

For the SMC that corresponds to VA 4, shown in Figure 6d, the reflection on the whiteboard (visible at angles <−1.5 rad) has very high SINR, much higher than reflections on the other materials, e.g., the wooden door and the plasterboard wall. From VA 6, c.f. Figure 6e, it is shown that reflections on the NGW with metal-coated glass windows yield very significant ranging information, reaching up to an SINR of around 20.

As shown in Figure 6f,g,i,k, for VA 7, 8, 11 and 13, the SINR is not as good as the first-order SMCs, but better than the other second-order SMCs shown in Figure 6h,j,l,m,n.

**Figure 7:**
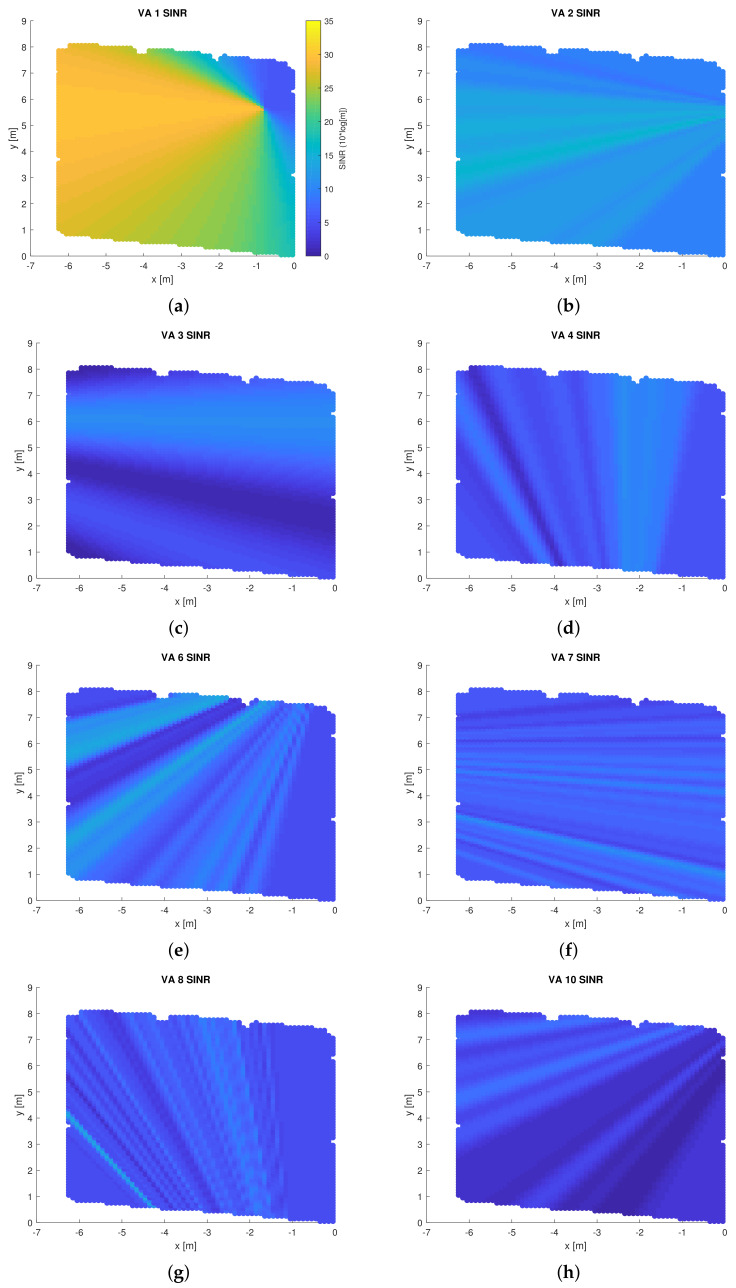

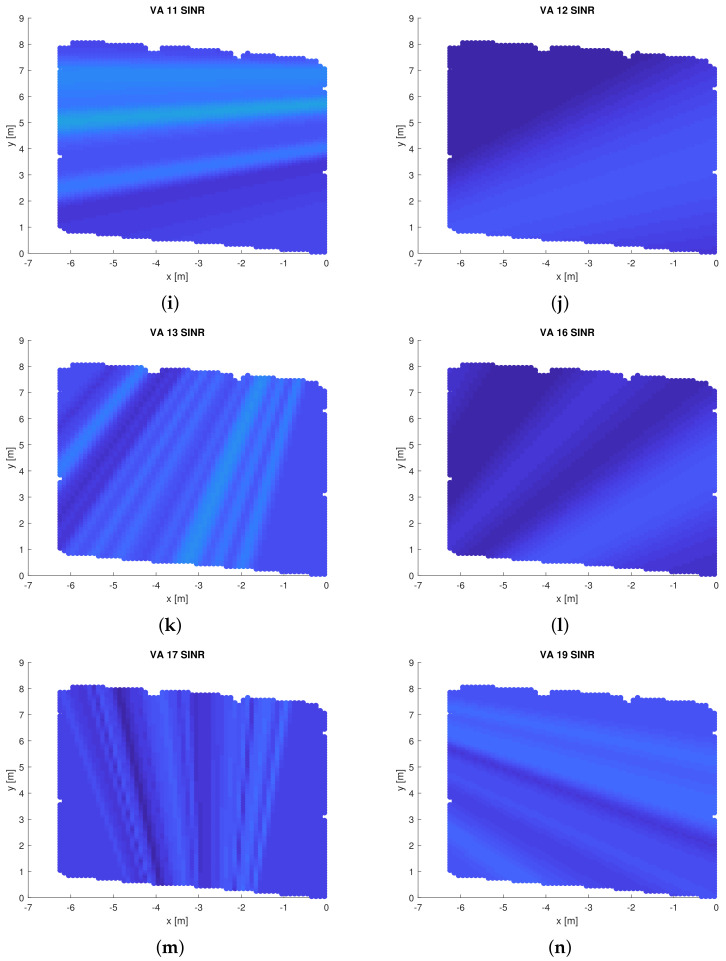
Predicted SINR for the whole floor plan for various links: (**a**) LOS, (**b**) reflection on EPB, (**c**) reflection on WW, (**d**) reflection on on south part of the room, (**e**) reflection on the north part of the room, (**f**) double reflection on EPB then WW, (**g**) double reflection on WW then SW, (**h**) double reflection on EPB then NGW, (**i**) double reflection on WW then EPB, (**j**) double reflection on WW then SW, (**k**) double reflection on SW then GW, (**l**) double reflection on SW then WW, (**m**) double reflection on NGW then SW, (**n**) double reflection on NGW then WW. SINR is obtained by applying GPR on the CIRs obtained from measurement at 595 points along the trajectory, as shown in Figure 1. The same dB scale is used in all sub figures.

**Figure 8:**
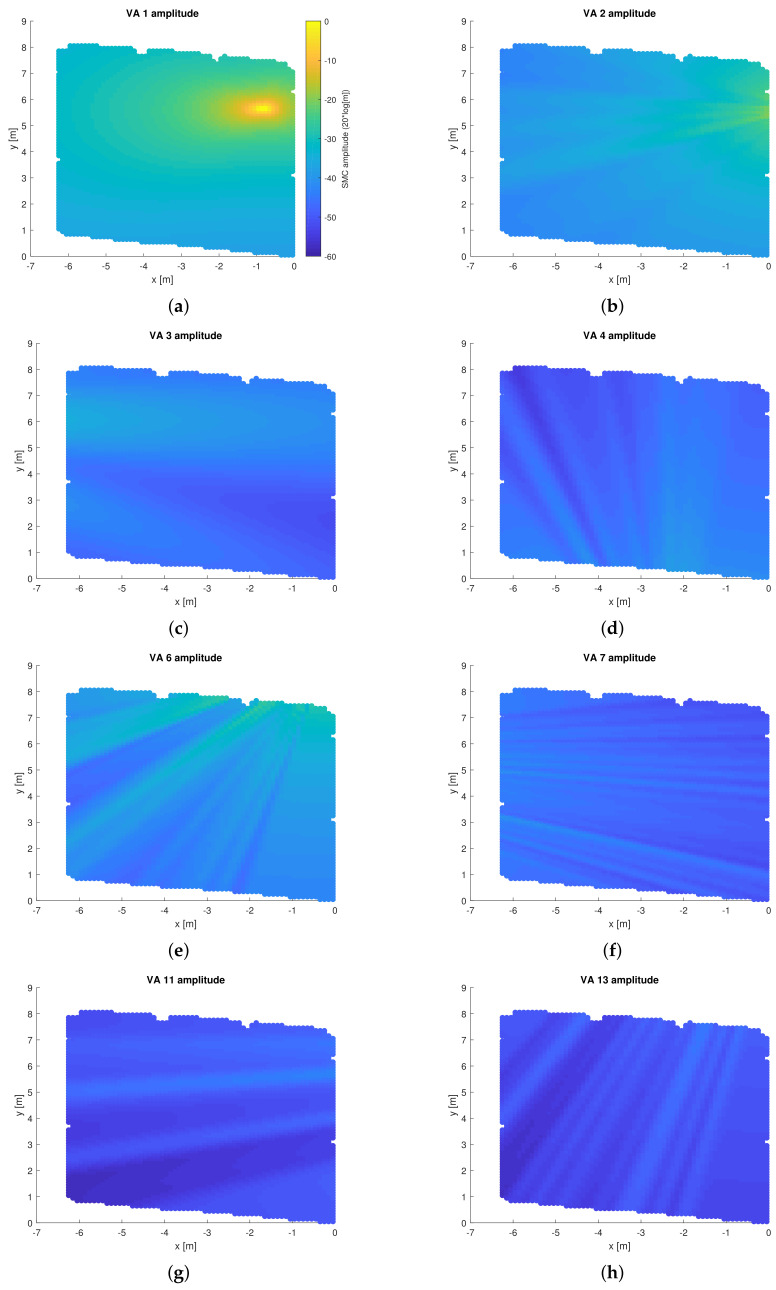
Predicted SMC amplitude $\alpha_{k}\left(p\right)$ for the whole floor plan for various SMCs: (**a**) LOS, (**b**) reflection on EPB, (**c**) reflection on WW, (**d**) reflection on on south part of the room, (**e**) reflection on the north part of the room, (**f**) double reflection on EPB then WW, (**g**) double reflection on WW then EPB, (**h**) double reflection on the SW then NGW. GPR was applied the set of measurement along the trajectory. The same dB scale is used in all sub-figures.

Implementing GPR on measurement data allows us to predict the SINR as well as the amplitude of the SMCs for each point $p^{*}$ in an environment at which an SMC is visible. Figure 7 shows this prediction result in terms of the ${\mathrm{SINR}}_{k}\left(p^{*}\right)$, while Figure 8 shows the predicted mean amplitude $E\left[\psi \left(\phi_{k}\left(p^{*}\right)\right)|D_{k},\theta_{k}^{\mathrm{abs}}\right]/∥a_{k}-p^{*}∥$. It is evident that the LOS path has very high SINR compared to other SMCs, thus it yields very high range information. However, in the case that the LOS is not available, choosing a good NLOS SMC is necessary. Two parameters could be used to choose between SMCs, the SINR and the SMC amplitude. The SINR is an appropriate measure to describe the ranging information provided by some SMC, which is an important metric for (multipath-assisted) positioning algorithms (see Section 5), while the amplitude translates to communication-performance metrics like channel capacity [53] and bit error rate.

From Figure 8, it is seen that the LOS amplitude is dependent on the distance as well as the antenna radiating pattern, while for other SMCs, the amplitudes are also affected by the reflection coefficients of the plane surfaces.

### 4.3. Position Error Bound

**Figure 9:**
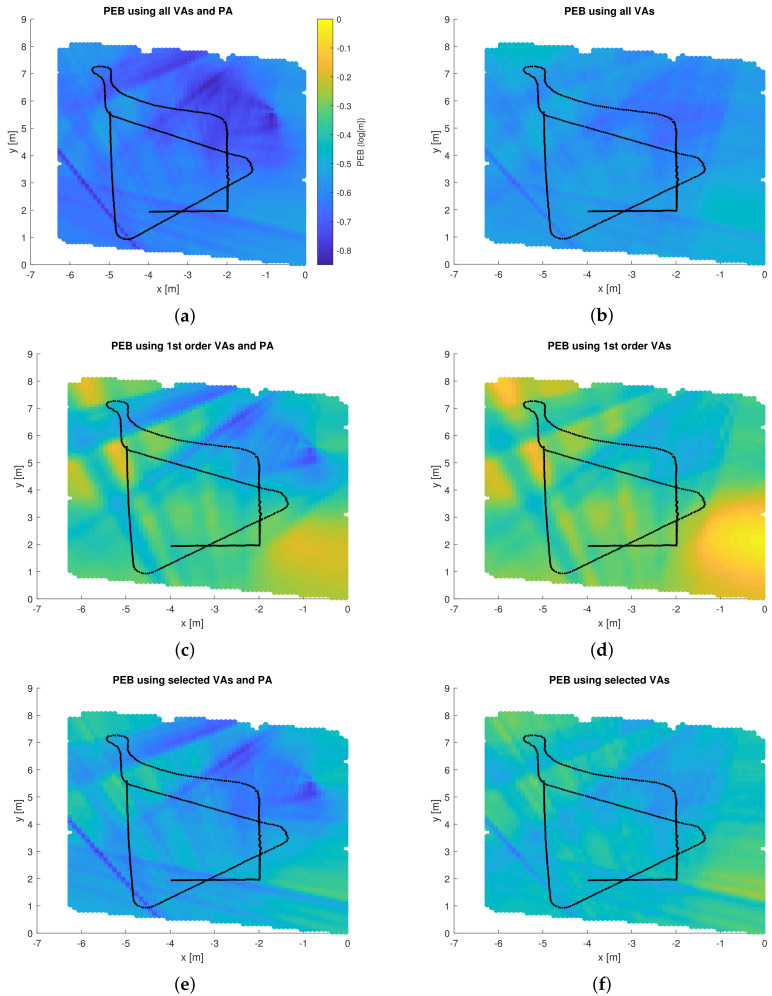
GPR-predicted PEB considering information from SMCs corresponding to: (**a**) all VAs and the PA, (**b**) all 1st and 2nd-order VAs, (**c**) 1st-order VAs and the PA, (**d**) all 1st-order VAs, (**e**) selected VAs and the LOS, (**f**) selected VAs.

Figure 9 shows the predicted PEB throughout the floor plan, computed from (5), when using information from various SMCs. The information provided by each SMC is quantified by ${\mathrm{SINR}}_{k}\left(p^{*}\right)$. The sub-figures on the left-hand side include the LOS component from the PA, while the LOS is neglected in all sub-figures on the right. In the top row, all VAs are considered, while first-order VAs only are considered in the middle, and at the bottom, only VA 2, VA 4, VA 6, VA 7, and VA 8 are used, which contribute most to the PEB.

It is observed that the second-order VAs play an important role in any localization algorithm, despite their low powers. With information from them, the PEB improves significantly even without the LOS component. With only first-order VAs, the PEB is worse.

## 5. Exploiting the GPR Model for (Multipath-Assisted) Positioning

To formulate a signal model for position estimation, Equation (3) can be rewritten as

(10) $$r=S\left(p\right)\alpha +w∼\mathrm{CN}\left(S(p)\alpha ,C\right)$$

where $r=\left[\ldots ,r(nT_{s}),\ldots \right]\in C^{N}$ is a sampled version of the received signal, $S\left(p\right)=\left[\ldots ,s(\tau_{k}\left(p\right)),\ldots \right]$ is the parameterized signal matrix with $s\left(\tau \right)={\left[\ldots ,s(nT_{s}-\tau ),\ldots \right]}^{T}\in C^{N}$, denoting the SMCs with delays $\tau_{k}\left(p\right)$ that are determined by p, $\alpha ={\left[\ldots ,\alpha_{k},\ldots \right]}^{T}\in C^{K}$ are the SMC amplitudes, and w is a noise vector with covariance $C=E\left\{ww^{H}\right\}$. The noise vector w may account for AWGN only or for the DM as well. A maximum likelihood (ML) estimation $\hat{p}=\arg\max_{p^{*}}L\left(r|p^{*}\right)$ considers α as nuisance parameters and finds the position by maximizing the concentrated log-likelihood function (LLHF)

(11) $$L\left(r|p^{*}\right)=\max_{\alpha }L\left(r|p^{*},\alpha \right).$$

The LLHF evaluated at candidate position $p^{*}$ follows from (10),

(12) $$L\left(r|p^{*},\alpha \right)=-\log\det\left(C\right)-{\left(r-S(p)\alpha \right)}^{H}C^{-1}\left(r-S(p)\alpha \right).$$

The maximization in (11) is solved by a weighted least square solution for ${∥r-S(p)\alpha ∥}^{2}$ which yields $\hat{\alpha }={\left(S^{H}\left(p\right)C^{-1}S\left(p\right)\right)}^{-1}S^{H}\left(p\right)C^{-1}r$. This ML positioning algorithm is our reference algorithm, denoted as “SALMA-light” and described in [54,55] if AWGN is assumed, i.e., $C=\sigma_{n}^{2}I$.

The GPM model provides prior knowledge about the absolute value of α but not on its phase. We thus expand the amplitude vector as α=Φx, where diagonal matrix Φ=diag(ϕ) and ${\left[\phi \right]}_{k}=\exp\left(j∠\alpha_{k}\right)=\exp\left(j\zeta_{k}\right)$. From the GPM, we know that $x|p∼N\left(\mu \left(p\right),\frac{1}{2}\Lambda \left(p\right)\right)$ with ${\left[\mu \left(p\right)\right]}_{k}=\frac{E\left\{\psi \left(\phi_{k}\left(p\right)\right)|D_{k},\theta_{k}^{\mathrm{abs}}\right\}}{\|a_{k}-p\|}$, and the diagonal matrix has elements ${\left[\Lambda \left(p\right)\right]}_{k,k}=2\frac{V\left\{\psi \left(\phi_{k}\left(p\right)\right)|D_{k},\theta_{k}^{\mathrm{abs}}\right\}}{{\|a_{k}-p\|}^{2}}$. The signal model then becomes

(13) $$r=S\left(p\right)\Phi \mu \left(p\right)+S\left(p\right)\tilde{x}+w$$

with $\tilde{x}|p∼\mathrm{CN}\left(0,\Lambda (p)\right)$ is introduced to account for the variance of the SMC amplitudes and the uncertainty of the phases in Φ.

The concentrated LLHF is now obtained by maximizing the LLHF for signal model (13) by maximizing for the nuisance parameter vector ϕ and $\sigma_{n}^{2}$ (maximizing the AWGN model for C), given candidate position $p^{*}$,

(14) $$F\left(p^{*}\right)=\max_{\sigma_{n},\phi }L\left(r|\sigma_{n}^{2},\phi ,p^{*}\right)$$

with

(15) $$L\left(r|\sigma_{n},\phi ,p^{*}\right)=-\log\det\left(\tilde{C}\right)-{\left(r-S(p)M(p)\phi \right)}^{H}{\tilde{C}}^{-1}\left(r-S(p)M(p)\phi \right)$$

where M(p)=diag(μ(p)) and

(16) $$\tilde{C}=\sigma_{n}^{2}I+S\left(p\right)\Lambda \left(p\right)S{\left(p\right)}^{H}$$

for the noise covariance.

First, we will find the phase ϕ, and noise variance $\sigma_{n}^{2}$ that maximize (15), i.e.,

(17) $$\hat{\phi }=\arg\max_{\phi }L\left(r|\sigma_{n},\phi ,p^{*}\right)$$

(18) $${\hat{\sigma }}_{n}=\arg\max_{\sigma_{n}}L\left(r|\sigma_{n}^{2},\hat{\phi },p^{*}\right)$$

Let’s denote the phase of the *k*-th SMC amplitude as $\zeta_{k}$. To solve for the phase, we have:

(19) $$\begin{array}{c} {\left(r-S(p)M(p)\phi \right)}^{H}{\tilde{C}}^{-1}\left(r-S(p)M(p)\phi \right)=\\ {\left(r-\sum_{k^{\prime }\neq k}s\left(\tau_{k}\right)\Lambda_{k}e^{j\zeta_{k}}-s\left(\tau_{k}\right)\mu_{k}e^{j\zeta_{k}}\right)}^{H}{\tilde{C}}^{-1}\left(r-\sum_{k^{\prime }\neq k}s\left(\tau_{k}\right)\mu_{k}e^{j\zeta_{k}}-s\left(\tau_{k}\right)\mu_{k}e^{j\zeta_{k}}\right)\\ ={\left(r_{k}-s\left(\tau_{k}\right)\mu_{k}e^{j\zeta_{k}}\right)}^{H}{\tilde{C}}^{-1}\left(r_{k}-s\left(\tau_{k}\right)\mu_{k}e^{j\zeta_{k}}\right)=f\left(\zeta_{k}\right) \end{array}$$

Vector $r_{k}$ is the residual of the CIR measurement after subtracting all SMCs except the *k*-th.

In order to find $\zeta_{k}$, let’s denote $e^{j\zeta_{k}}=x_{k}+jy_{k}$. Thus:

(20) $${\hat{x}}_{k},{\hat{y}}_{k}=\arg\min_{x_{k},y_{k}}f\left(x_{k},y_{k}\right)\;s.t.\;g\left(x_{k},y_{k}\right)=x_{k}^{2}+y_{k}^{2}-1=0$$

Using Lagrange multiplier, we have

(21) $${\hat{x}}_{k},{\hat{y}}_{k},{\hat{\lambda }}_{k}=\arg\min_{x_{k},y_{k},\lambda_{k}}f\left(x_{k},y_{k}\right)+\lambda_{k}g\left(x_{k},y_{k}\right)$$

and obtain

(22) ∂L∂xk=−2μks(τk)HC˜−1Rerk+2xkμk2s(τk)HC˜−1s(τk)+2xkλk

(23) ∂L∂yk=−2μks(τk)HC˜−1Imrk+2ykμk2s(τk)HC˜−1s(τk)+2ykλk

(24) $$\frac{\partial L}{\partial \lambda k}=x_{k}^{2}+y_{k}^{2}-1$$

Equating Equations (22)–(24) to 0, we have:

(25) $$\lambda_{k}=\left|s^{H}\left(\tau_{k}\right){\tilde{C}}^{-1}\mu_{k}r_{k}\right|-\mu_{k}^{2}s{\left(\tau_{k}\right)}^{H}{\tilde{C}}^{-1}s\left(\tau_{k}\right)$$

and

(26) xk=s(τk)HC˜−1μkRe{rk}μk2s(τk)HC˜−1s(τk)+λk

Similarly for $y_{k}$. Or, in short we have

(27) $$\zeta_{k}=∠s{\left(\tau_{k}\right)}^{H}{\tilde{C}}^{-1}\mu_{k}r_{k}$$

and if the *k*-th SMC does not overlap with any other SMC, we have equivalently

(28) $${\hat{\zeta }}_{k}=∠s\left(\tau_{k}\right){\tilde{C}}^{-1}r$$

which is a weighted projection of the measurement on the *k*-th SMC template $s(\tau_{k})$. The weighting (by ${\tilde{C}}^{-1}$) takes into account the variance of the SMC from the GPR model, which is essential for reducing secondary maxima in the LHF. Unfortunately, the weighting has a negative influence on the reliability of this phase estimation step. We thus replace for this estimator the covariance by an identity matrix, obtaining the approximate (but more robust) phase estimator

(29) $${\hat{\zeta }}_{k}\approx ∠s\left(\tau_{k}\right)r.$$

From (15), we can write:

(30) $$L\left(r|\sigma_{n},\phi ,p^{*}\right)=-\log\det\tilde{C}-\mathrm{trace}\left\{{\tilde{C}}^{-1}\hat{\tilde{C}}\right\}$$

where $\hat{\tilde{C}}=\left(r-S(p)M(p)\phi \right){\left(r-S(p)M(p)\phi \right)}^{H}$ is a realization of $\tilde{C}$. According to [56], we have:

(31) $${\hat{\sigma_{n}}}^{2}=\frac{1}{N-K}\mathrm{trace}\left\{\Pi_{S(\tau )}^{⊥}\hat{\tilde{C}}\right\}$$

in which

(32) $$\Pi_{S(\tau )}^{⊥}=I_{N}-S\left(\tau \right){\left(S{\left(\tau \right)}^{H}S\left(\tau \right)\right)}^{-1}S{\left(\tau \right)}^{H}.$$

Thus ${\hat{\sigma }}_{n}$ can be computed. The localization problem reduces to

(33) $$\hat{p}=\arg\max_{p^{*}}L\left(r|{\hat{\sigma }}_{n},\hat{\phi },p^{*}\right).$$

## 6. Result

**Figure 10:**
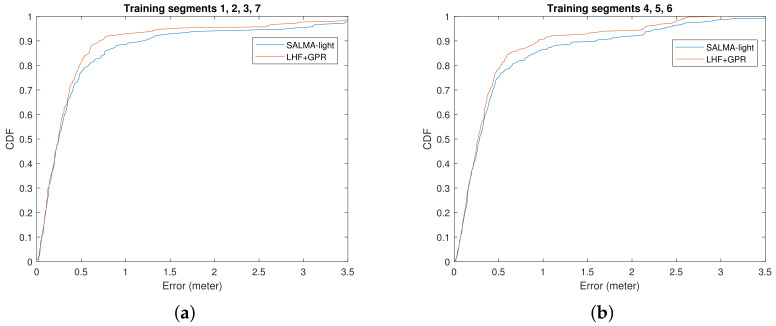
CDF of the localization error using (**a**) training segments 1, 2, 3, 7, (**b**) training segments 4, 5, 6. The blue solid line plots the error CDF obtained when SALMA-light is used. The orange solid line plots the error CDF obtained when optimizing the LLHF with prior knowledge from GPR. The phase $\zeta_{k}$ is approximated by using the phase from CIR. Overlap checking is performed first before implementing the algorithms.

To implement the GPR-based positioning algorithm, the data described in Section 3 is divided into two groups. The first group is used for GP training, in which both the agent positions and the corresponding CIRs are known. The GPM hyper-parameters are estimated. The second group contains the remaining data, where only the CIRs are used. The corresponding agent positions are unknown and will be estimated by exploiting the REM, i.e., the SMC amplitudes and SINRs predicted using the GPM hyper-parameters. Errors are computed and their CDFs are plotted in Figure 10. SALMA-light [54,55], the reference single-anchor localization method described in Section 5, is used for comparison.

It is shown that, with situational awareness through the REM information, the proposed algorithm outperforms SALMA-light in terms of the outlier robustness, while the accuracy remains comparable.

**Figure 11:**
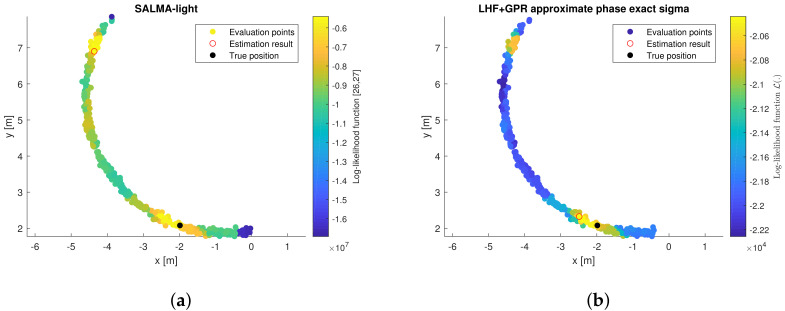
LLHF values obtained using (**a**) SALMA-light, (**b**) LHF+GPR with exact sigma. LLHF values at evaluation points are plotted with color code. The red circle denotes the estimation result, while the filled black circle denotes the true position.

To explain this performance improvement, Figure 11 compares the LLHF for the two algorithms. The LLHF has been evaluated at candidate positions lying on a radius that corresponds to the estimated line-of-sight distance [55]. It is shown that the REM-assisted algorithm reduces the number of local maxima in the LLHF and emphasizes the correct maximum, yielding the performance improvement over SALMA-light.

**Figure 12:**
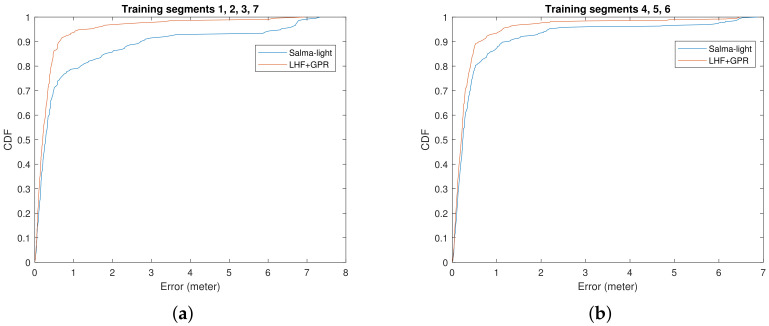
CDF of the localization error using (**a**) training segments 1, 2, 3, 7, (**b**) training segments 4, 5, 6. The blue solid line plots the error CDF obtained when SALMA-light is used. The orange solid line plots the error CDF obtained when optimizing the LLHF with prior knowledge from GPR. The phase $\zeta_{k}$ is approximated by using the phase from CIR. Visibility test is carried out, followed by overlap checking, before implementing the algorithms.

Figure 12 shows the error CDF for a modified experiment, where a visibility test and overlap checking are conducted to reduce the number of SMCs that are used for position estimation to the most useful ones. This is done for both, SALMA-light and the proposed algorithm. It is shown that again the proposed algorithm outperforms SALMA-light in reducing outliers. The difference in results between Figure 10 and Figure 12 is because, after visibility test and overlap checking, the number of VAs used is reduced, leading to reduced robustness for SALMA light while the use of the REM avoids such loss of robustness. Note that also the accuracy is improved in this variant.

These results suggest that the proposed algorithm might perform better in obstructed-LOS or in the presence of humans or obstacles, even though the algorithm has not been tailored to specifically address this problem. The recent publications [57,58] describe algorithms that are designed to be robust to blocked LOS (in the former) and blocked SMCs (in the latter), in particular when the blocking is due to the influence of the human body on the visibility of SMCs. The belief propagation SLAM algorithm from [38] provides visibility information of SMCs in addition to the amplitude and delay information. This visibility information can be used to pre-select the data points for the construction of the GPR-based REM such that path blocking does not influence the quality of the REM. It is expected that the “environment part” of the propagation channel, which is expressed by the GPR-based REM, and the “device part” can be separated very well, once a consistent REM has been learned from a large number of independent measurements obtained from different user equipment. The rigorous formulation of such methods is left for future work, however.

## 7. Conclusions

In this paper, radio environment maps (REMs) have been formulated, taking into account amplitude information of specular multipath components (SMCs) that are learned by means of Gaussian process regression (GPR). The regression method allows predicting the mean and variance of the SMC amplitudes. From these parameters, the position-error-bound (PEB) has been computed as a performance metric for positioning algorithms that exploit range information contained in the SMCs, so-called multipath-based localization schemes.

The amplitude information can also be used to formulate REMs of communication performance metrics, e.g., the channel capacity or the bit-error rate, in order to realize more efficient and robust, location-aware protocols for medium access control. Using a set of training measurements from the environment, the REMs can be computed for any point within the environment. The paper evaluates to what extent the REM represents the multipath components in the analyzed data. It has been found that 50% to 90% of the received power can be attributed to the resolvable SMCs contained in our model, which corresponds to the fraction of power that can be predicted by the REM.

The REM is applied in this paper to improve the robustness of a single-anchor multipath-based localization method which sometimes suffers from outliers resulting in large errors. Using the REM as a channel prior knowledge helps to avoid these outliers.

## Data Availability

The measurement data are available on request from the authors, c.f. https://www.spsc.tugraz.at/databases-and-tools/uwb-indoor-channel-experimental-data.html.
